# Supplementary material for: A Standardized Extract of Microalgae Phaeodactylum tricornutum (Mi136) Inhibit D-Gal Induced Cognitive Dysfunction in Mice
Source: Mar Drugs. 2024 Feb 21;22(3):99. doi: 10.3390/md22030099 (PMC10972078; doi:10.3390/md22030099)
Supplement: Supplementary file 1 [file marinedrugs-22-00099-s001.zip › marinedrugs-2871900-supplementary.pdf]

Supplementary material

Table S1. Raw data on behavorial tests in blank control group (BC) : Y-maze test ; Passive avoidance test and Morris water maze test.

Abbreviations: ALT (Alternation) ; STL (Step through latency) ; EL (Escape Latency) ; Lat (Latency) ; Med (Median) ; Tg (Target quadrant)

		Y-MAZE			STPA Task		MORRIS WATER MAZE TEST																					
							DAY 01				DAY 02				DAY 03				DAY 04				DAY 05				Probe test	
#Gp	# Mouse	LOC	ALT	%ALT	STL	EL	Lat1	Lat2	Lat3	*Med*	Lat1	Lat2	Lat3	*Med*	Lat1	Lat2	Lat3	*Med*	Lat1	Lat2	Lat3	*Med*	Lat1	Lat2	Lat3	*Med*	*Tg*	*Other*
BC	617-B1	33	25	80,6	179	37	66	59	90	*66*	63	90	24	*63*	90	38	26	*38*	90	27	19	*27*	24	13	39	*24*	*40,2*	*19,9*
	617-B2	27	19	76,0	226	8	90	90	90	*90*	90	68	53	*68*	36	13	84	*36*	90	30	34	*34*	10	77	15	*15*	*53,9*	*15,4*
	617-B3	37	29	82,9	300	14	90	90	58	*90*	56	90	55	*56*	90	28	41	*41*	39	45	18	*39*	7	8	90	*8*	*36,4*	*21,2*
	617-B4	34	13	40,6	213	39	59	67	90	*67*	27	90	49	*49*	26	90	10	*26*	90	26	13	*26*	17	75	20	*20*	*51,5*	*16,2*
	617-B5	34	21	65,6	300	27	90	75	90	*90*	68	70	90	*70*	15	90	29	*29*	6	90	15	*15*	90	29	13	*29*	*53,1*	*15,6*
	617-B6	33	15	48,4	300	12	90	90	74	*90*	62	90	52	*62*	78	30	23	*30*	30	15	90	*30*	90	11	12	*12*	*54,5*	*15,2*
	617-J1	29	20	74,1	300	22	90	90	47	*90*	65	90	14	*65*	36	90	9	*36*	90	26	8	*26*	35	27	21	*27*	*43*	*19*
	617-J2	33	22	71,0	224	15	62	90	39	*62*	90	59	39	*59*	90	44	27	*44*	30	90	27	*30*	17	90	35	*35*	*46,3*	*17,9*
	617-J3	37	23	65,7	300	19	90	47	90	*90*	90	27	47	*47*	90	10	30	*30*	90	19	12	*19*	10	77	6	*10*	*42,1*	*19,3*
	617-J4	34	21	65,6	237	7	90	69	51	*69*	62	60	56	*60*	42	32	90	*42*	90	40	47	*47*	90	8	5	*8*	*40,4*	*19,9*
	617-J5	31	22	75,9	300	36	90	53	90	*90*	90	70	34	*70*	27	54	90	*54*	20	23	90	*23*	90	14	16	*16*	*32,2*	*22,6*
	617-J6	30	18	64,3	201	14	90	90	90	*90*	90	29	49	*49*	90	15	48	*48*	36	34	90	*36*	13	10	80	*13*	*42,4*	*19,2*
	**Mean**	**33**	**21**	**67,56**	**256,67**	**20,83**	**83,1**	**75,8**	**74,9**	**82**	**71,1**	**69,4**	**46,8**	**59,8**	**59,2**	**44,5**	**42,3**	**37,8**	**58,4**	**38,8**	**38,6**	**29,3**	**41,1**	**36,6**	**29,3**	**18,1**	**44,7**	**18,44**
	**SD**	**3**	**4,2**	**12,48**	**47,385**	**11,38**	**12,6**	**16,4**	**20,3**	**11,9**	**19,6**	**22,9**	**18,9**	**8,14**	**31**	**30,2**	**29,7**	**8,41**	**34**	**25,3**	**32,8**	**8,8**	**36,9**	**32,8**	**28**	**8,89**	**7,25**	**2,418**

Table S2. Raw data on behavorial tests in negative control group (NC) : Y-maze test ; Passive avoidance test and Morris water maze test.

Abbreviations: ALT (Alternation) ; STL (Step through latency) ; EL (Escape Latency) ; Lat (Latency) ; Med (Median) ; Tg (Target quadrant)

		Y-MAZE			STPA Task		MORRIS WATER MAZE TEST																					
							DAY 01				DAY 02				DAY 03				DAY 04				DAY 05				Probe test	
#Gp	# Mouse	LOC	ALT	%ALT	STL	EL	Lat1	Lat2	Lat3	*Med*	Lat1	Lat2	Lat3	*Med*	Lat1	Lat2	Lat3	*Med*	Lat1	Lat2	Lat3	*Med*	Lat1	Lat2	Lat3	*Med*	*Tg*	*Other*
NC	617-D1	35	10	30,3	108	68	90	90	90	*90*	88	90	43	*88*	90	77	48	*77*	90	66	23	*66*	63	84	13	*63*	*18,2*	*27,3*
	617-D2	29	10	37,0	79	77	90	90	63	*90*	54	90	36	*54*	56	90	44	*56*	47	90	15	*47*	22	41	90	*41*	*26,3*	*24,6*
	617-D3	32	12	40,0	147	124	59	47	90	*59*	90	70	26	*70*	90	37	49	*49*	36	59	90	*59*	38	90	8	*38*	*30,2*	*23,3*
	617-D4	29	15	55,6	136	82	90	66	90	*90*	90	90	90	*90*	90	67	65	*67*	54	90	62	*62*	90	15	27	*27*	*32,4*	*22,5*
	617-D5	38	17	47,2	84	108	90	90	90	*90*	90	90	27	*90*	59	90	24	*59*	90	38	42	*42*	90	75	19	*75*	*18,5*	*27,2*
	617-D6	31	11	37,9	190	94	90	54	74	*74*	69	90	73	*73*	63	90	70	*70*	59	90	90	*90*	90	90	90	*90*	*29,6*	*23,5*
	617-L1	35	14	42,4	124	79	37	59	90	*59*	66	37	90	*66*	90	66	17	*66*	66	90	13	*66*	90	62	51	*62*	*17,3*	*27,6*
	617-L2																											
	617-L3	32	11	36,7	109	87	90	47	66	*66*	90	59	19	*59*	75	90	39	*75*	90	59	22	*59*	36	90	24	*36*	*35*	*21,7*
	617-L4	28	12	46,2	86	130	90	90	51	*90*	90	90	90	*90*	90	84	90	*90*	44	90	19	*44*	90	49	17	*49*	*22,3*	*25,9*
	617-L5	34	14	43,8	137	75	90	90	90	*90*	90	90	90	*90*	90	40	36	*40*	40	63	90	*63*	51	67	38	*51*	*16,7*	*27,8*
	617-L6	30	10	35,7	172	69	48	70	90	*70*	65	46	90	*65*	90	60	27	*60*	36	90	37	*37*	22	90	39	*39*	*21,2*	*26,3*
	**Mean**	**32**	**12**	**41,16**	**124,73**	**90,27**	**78,5**	**72,1**	**80,4**	**78,9**	**80,2**	**76,5**	**61,3**	**75,9**	**80,3**	**71,9**	**46,3**	**64,5**	**59,3**	**75**	**45,7**	**57,7**	**62**	**68,5**	**37,8**	**51,9**	**24,3**	**25,22**
	**SD**	**3,1**	**2,3**	**6,883**	**36,119**	**21,47**	**20,2**	**18,4**	**14,4**	**13,4**	**13,7**	**20,3**	**30,7**	**14**	**14,2**	**19,8**	**21,7**	**13,9**	**21,8**	**18,6**	**31,7**	**14,9**	**29,1**	**24,8**	**28,7**	**18,9**	**6,62**	**2,207**

Table S3. Raw data on behavorial tests in BP120 group : Y-maze test ; Passive avoidance test and Morris water maze test.

Abbreviations: ALT (Alternation) ; STL (Step through latency) ; EL (Escape Latency) ; Lat (Latency) ; Med (Median) ; Tg (Target quadrant)

		Y-MAZE			STPA Task		MORRIS WATER MAZE TEST																					
							DAY 01				DAY 02				DAY 03				DAY 04				DAY 05				Probe test	
#Gp	# Mouse	LOC	ALT	%ALT	STL	EL	Lat1	Lat2	Lat3	*Med*	Lat1	Lat2	Lat3	*Med*	Lat1	Lat2	Lat3	*Med*	Lat1	Lat2	Lat3	*Med*	Lat1	Lat2	Lat3	*Med*	*Tg*	*Other*
BP120	617-F1	32	17	56,7	187	74	90	90	90	*90*	90	84	29	*84*	90	55	24	*55*	90	40	17	*40*	90	47	14	*47*	*37,5*	*20,8*
	617-F2	31	19	65,5	235	39	90	90	90	*90*	67	90	40	*67*	48	37	90	*48*	37	90	13	*37*	38	90	18	*38*	*37,9*	*20,7*
	617-F3	37	20	57,1	174	26	75	40	90	*75*	54	59	90	*59*	10	37	90	*37*	90	28	19	*28*	90	51	21	*51*	*48,7*	*17,1*
	617-F4	29	14	51,9	159	47	90	90	59	*90*	36	90	73	*73*	90	22	39	*39*	90	22	50	*50*	13	75	29	*29*	*47,5*	*17,5*
	617-F5	33	19	61,3	242	31	90	90	63	*90*	90	75	74	*75*	52	75	19	*52*	60	40	30	*40*	22	34	90	*34*	*29*	*23,7*
	617-F6	31	18	62,1	207	60	24	90	41	*41*	90	29	70	*70*	90	27	30	*30*	22	54	10	*22*	42	90	18	*42*	*27,8*	*24,1*
	617-N1	28	14	53,8	224	47	90	90	90	*90*	90	74	70	*74*	90	50	27	*50*	90	47	42	*47*	90	36	24	*36*	*35,3*	*21,6*
	617-N2	35	19	57,6	256	36	90	90	90	*90*	39	90	27	*39*	44	90	15	*44*	38	90	30	*38*	41	90	19	*41*	*49,2*	*16,9*
	617-N3	31	15	51,7	170	51	90	55	69	*69*	34	65	90	*65*	90	39	28	*39*	90	51	40	*51*	15	29	56	*29*	*38,8*	*20,4*
	617-N4	38	19	52,8	197	43	90	90	77	*90*	56	45	90	*56*	90	90	32	*90*	90	24	26	*26*	90	22	37	*37*	*40,2*	*19,9*
	617-N5																											
	617-N6	33	18	58,1	200	29	90	39	46	*46*	90	27	79	*79*	52	90	41	*52*	40	19	90	*40*	18	7	90	*18*	*43*	*19*
	**Mean**	**33**	**17**	**57,14**	**204,64**	**43,91**	**82,6**	**77,6**	**73,2**	**78,3**	**66,9**	**66,2**	**66,5**	**67,4**	**67,8**	**55,6**	**39,5**	**48,7**	**67**	**45,9**	**33,4**	**38,1**	**49,9**	**51,9**	**37,8**	**36,5**	**39,5**	**20,15**
	**SD**	**3,1**	**2,2**	**4,474**	**31,541**	**14,25**	**20**	**21,6**	**18,8**	**18,7**	**24**	**23,6**	**23,7**	**12,5**	**27,8**	**26,2**	**26,1**	**15,7**	**27,8**	**24,8**	**22,6**	**9,52**	**33,3**	**29,9**	**28,3**	**9,14**	**7,26**	**2,42**

Supplementary material

Table S4. Raw data on behavorial tests in BP235 group : Y-maze test ; Passive avoidance test and Morris water maze test.

Abbreviations: ALT (Alternation) ; STL (Step through latency) ; EL (Escape Latency) ; Lat (Latency) ; Med (Median) ; Tg (Target quadrant)

		Y-MAZE			STPA Task		MORRIS WATER MAZE TEST																					
							DAY 01				DAY 02				DAY 03				DAY 04				DAY 05				Probe test	
#Gp	# Mouse	LOC	ALT	%ALT	STL	EL	Lat1	Lat2	Lat3	*Med*	Lat1	Lat2	Lat3	*Med*	Lat1	Lat2	Lat3	*Med*	Lat1	Lat2	Lat3	*Med*	Lat1	Lat2	Lat3	*Med*	*Tg*	*Other*
BP235	617-H1	30	23	82,1	300	20	90	90	90	*90*	54	90	41	*54*	90	30	17	*30*	90	26	12	*26*	37	22	10	*22*	*46,2*	*17,9*
	617-H2	35	25	75,8	300	17	90	90	90	*90*	37	60	90	*60*	27	90	20	*27*	15	90	8	*15*	19	26	13	*19*	*39,6*	*20,1*
	617-H3	31	24	82,8	179	22	55	90	39	*55*	46	90	49	*49*	13	90	36	*36*	90	9	10	*10*	48	9	30	*30*	*37,6*	*20,8*
	617-H4	29	22	81,5	300	9	74	90	28	*74*	59	63	90	*63*	59	46	28	*46*	90	12	13	*13*	90	17	20	*20*	*30,7*	*23,1*
	617-H5	35	23	69,7	241	35	90	77	90	*90*	73	90	70	*73*	29	70	15	*29*	47	28	13	*28*	24	37	14	*24*	*48,3*	*17,2*
	617-H6	32	22	73,3	208	40	82	90	40	*82*	90	42	48	*48*	50	52	35	*50*	25	83	19	*25*	90	6	12	*12*	*40,7*	*19,8*
	617-P1	31	21	72,4	300	17	90	90	90	*90*	55	90	54	*55*	90	27	13	*27*	90	27	26	*27*	42	55	62	*55*	*39,8*	*20,1*
	617-P2	29	14	51,9	213	41	90	82	74	*82*	90	39	18	*39*	43	90	42	*43*	15	12	90	*15*	70	17	18	*18*	*26,1*	*24,6*
	617-P3	39	28	75,7	300	9	77	64	90	*77*	74	90	70	*74*	90	55	53	*55*	90	19	18	*19*	7	39	6	*7*	*43,9*	*18,7*
	617-P4	33	22	71,0	300	23	90	90	90	*90*	90	56	15	*56*	39	90	28	*39*	90	9	27	*27*	48	84	21	*48*	*25,4*	*24,9*
	617-P5	30	21	75,0	249	17	82	37	90	*82*	29	90	36	*36*	30	90	36	*36*	20	30	90	*30*	10	24	47	*24*	*29,1*	*23,6*
	617-P6	37	24	68,6	186	39	90	90	90	*90*	90	31	64	*64*	37	40	90	*40*	24	90	13	*24*	62	39	12	*39*	*26,8*	*24,4*
	**Mean**	**33**	**22**	**73,3**	**256,33**	**24,08**	**83,3**	**81,7**	**75,1**	**82,7**	**65,6**	**69,3**	**53,8**	**55,9**	**49,8**	**64,2**	**34,4**	**38,2**	**57,2**	**36,3**	**28,3**	**21,6**	**45,6**	**31,3**	**22,1**	**26,5**	**36,2**	**21,27**
	**SD**	**3,3**	**3,3**	**8,261**	**49,425**	**11,71**	**10,6**	**16,2**	**24,4**	**10,4**	**22**	**23,4**	**24,4**	**11,9**	**26,9**	**25,3**	**21,2**	**9,15**	**35,2**	**31,9**	**29,4**	**6,8**	**28,3**	**21,8**	**16,7**	**14,3**	**8,2**	**2,734**

Table S5. Raw data on behavorial tests in BP295 group : Y-maze test ; Passive avoidance test and Morris water maze test.

Abbreviations: ALT (Alternation) ; STL (Step through latency) ; EL (Escape Latency) ; Lat (Latency) ; Med (Median) ; Tg (Target quadrant)

		Y-MAZE			STPA Task		MORRIS WATER MAZE TEST																					
							DAY 01				DAY 02				DAY 03				DAY 04				DAY 05				Probe test	
#Gp	# Mouse	LOC	ALT	%ALT	STL	EL	Lat1	Lat2	Lat3	*Med*	Lat1	Lat2	Lat3	*Med*	Lat1	Lat2	Lat3	*Med*	Lat1	Lat2	Lat3	*Med*	Lat1	Lat2	Lat3	*Med*	*Tg*	*Other*
BP295	617-A1	29	17	63,0	300	27	90	90	90	*90*	90	57	38	*57*	90	35	27	*35*	90	21	13	*21*	15	90	7	*15*	*45,4*	*18,2*
	617-A2	32	24	80,0	300	13	67	90	90	*90*	68	90	29	*68*	42	90	12	*42*	17	45	18	*18*	90	22	13	*22*	*39,1*	*20,3*
	617-A3	31	22	75,9	214	9	90	90	90	*90*	90	49	13	*49*	90	28	18	*28*	90	9	40	*40*	6	19	67	*19*	*52,2*	*15,9*
	617-A4	29	24	88,9	290	36	90	80	46	*80*	51	26	90	*51*	15	90	37	*37*	90	17	23	*23*	90	5	8	*8*	*51,2*	*16,3*
	617-A5	34	23	71,9	183	14	84	90	90	*90*	90	67	19	*67*	90	45	22	*45*	64	36	28	*36*	13	11	70	*13*	*33,6*	*22,1*
	617-A6	30	22	78,6	227	29	90	77	80	*80*	90	46	74	*74*	21	19	90	*21*	19	90	8	*19*	56	22	30	*30*	*43,6*	*18,8*
	617-I1	31	18	62,1	300	17	90	75	36	*75*	90	64	17	*64*	90	45	37	*45*	90	22	25	*25*	90	15	13	*15*	*57,4*	*14,2*
	617-I2	28	18	69,2	300	9	90	90	90	*90*	90	39	55	*55*	38	90	19	*38*	90	30	19	*30*	27	90	9	*27*	*52,3*	*15,9*
	617-I3	35	23	69,7	175	24	87	36	74	*74*	40	90	37	*40*	90	51	28	*51*	17	90	13	*17*	90	12	8	*12*	*41,8*	*19,4*
	617-I4	29	21	77,8	300	13	90	90	90	*90*	90	18	39	*39*	90	37	49	*49*	74	34	30	*34*	90	15	28	*28*	*35,8*	*21,4*
	617-I5	32	21	70,0	247	12	90	90	90	*90*	90	67	60	*67*	44	90	22	*44*	90	15	22	*22*	13	19	56	*19*	*53,6*	*15,5*
	617-I6	34	22	68,8	206	39	59	90	60	*60*	70	54	90	*70*	15	90	29	*29*	19	90	18	*19*	30	49	12	*30*	*30,7*	*23,1*
	**Mean**	**31**	**21**	**72,97**	**253,5**	**20,17**	**84,8**	**82,3**	**77,2**	**83,3**	**79,1**	**55,6**	**46,8**	**58,4**	**59,6**	**59,2**	**32,5**	**38,7**	**62,5**	**41,6**	**21,4**	**25,3**	**50,8**	**30,8**	**26,8**	**19,8**	**44,7**	**18,43**
	**SD**	**2,3**	**2,4**	**7,611**	**50,305**	**10,5**	**10,5**	**15,7**	**19,4**	**9,7**	**17,8**	**22,2**	**27,1**	**11,8**	**33,1**	**28,4**	**20,7**	**9,12**	**33,8**	**30,8**	**8,68**	**7,77**	**36,7**	**29,7**	**24**	**7,54**	**8,72**	**2,907**

Table S6. Raw data on behavorial tests in BP370 group : Y-maze test ; Passive avoidance test and Morris water maze test.

Abbreviations: ALT (Alternation) ; STL (Step through latency) ; EL (Escape Latency) ; Lat (Latency) ; Med (Median) ; Tg (Target quadrant)

		Y-MAZE			STPA Task		MORRIS WATER MAZE TEST																					
							DAY 01				DAY 02				DAY 03				DAY 04				DAY 05				Probe test	
#Gp	# Mouse	LOC	ALT	%ALT	STL	EL	Lat1	Lat2	Lat3	*Med*	Lat1	Lat2	Lat3	*Med*	Lat1	Lat2	Lat3	*Med*	Lat1	Lat2	Lat3	*Med*	Lat1	Lat2	Lat3	*Med*	*Tg*	*Other*
BP370	617-E1	35	26	78,8	300	27	42	38	90	*42*	90	67	60	*67*	90	37	17	*37*	90	27	13	*27*	17	66	15	*17*	*58,5*	*13,8*
	617-E2	31	23	79,3	217	13	90	90	90	*90*	53	90	47	*53*	48	24	75	*48*	13	90	10	*13*	8	29	39	*29*	*39,2*	*20,3*
	617-E3	29	21	77,8	179	29	90	90	26	*90*	90	38	49	*49*	90	36	30	*36*	67	36	27	*36*	15	55	10	*15*	*53,9*	*15,4*
	617-E4	33	27	87,1	237	43	27	75	90	*75*	56	61	90	*61*	90	18	29	*29*	90	10	18	*18*	70	12	13	*13*	*40,1*	*20*
	617-E5	37	26	74,3	300	17	30	69	90	*69*	70	90	72	*72*	35	42	27	*35*	41	58	15	*41*	13	26	86	*26*	*40,5*	*19,8*
	617-E6	27	19	76,0	300	30	90	34	90	*90*	38	90	54	*54*	13	90	24	*24*	90	8	13	*13*	40	16	30	*30*	*57,9*	*14*
	617-M 1	29	17	63,0	300	37	90	38	74	*74*	90	53	31	*53*	90	32	27	*32*	24	90	21	*24*	48	17	10	*17*	*32,8*	*22,4*
	617-M2	32	21	70,0	217	24	75	90	39	*75*	64	90	28	*64*	29	90	15	*29*	90	30	36	*36*	22	90	15	*22*	*35,6*	*21,5*
	617-M3	27	19	76,0	243	12	90	47	28	*47*	90	49	46	*49*	90	19	46	*46*	90	13	12	*13*	55	15	13	*15*	*53,7*	*15,4*
	617-M4	39	27	73,0	300	37	90	90	90	*90*	90	13	38	*38*	60	90	36	*60*	29	64	20	*29*	9	90	29	*29*	*31,8*	*22,7*
	617-M5	31	21	72,4	186	40	82	90	90	*90*	77	64	90	*77*	15	37	90	*37*	34	46	90	*46*	34	18	73	*34*	*56,7*	*14,4*
	617-M6	33	21	67,7	213	18	90	90	90	*90*	50	57	90	*57*	20	90	34	*34*	90	30	32	*32*	90	10	12	*12*	*44,5*	*18,5*
	**Mean**	**32**	**22**	**74,61**	**249,33**	**27,25**	**73,8**	**70,1**	**73,9**	**76,8**	**71,5**	**63,5**	**57,9**	**57,8**	**55,8**	**50,4**	**37,5**	**37,3**	**62,3**	**41,8**	**25,6**	**27,3**	**35,1**	**37**	**28,8**	**21,6**	**45,4**	**18,19**
	**SD**	**3,8**	**3,4**	**6,187**	**48,05**	**10,65**	**25,3**	**23,9**	**26,4**	**17,1**	**19**	**24,1**	**22,7**	**10,9**	**32,8**	**30,1**	**22,8**	**9,85**	**31,5**	**28,5**	**21,9**	**11,4**	**26,3**	**30,2**	**25,6**	**7,68**	**10,1**	**3,375**
